# Patient radiation exposure during general fluoroscopy examinations

**DOI:** 10.1120/jacmp.v15i2.4555

**Published:** 2014-03-06

**Authors:** Jeska S. Wambani, Geoffrey K. Korir, Mark A. Tries, Ian K. Korir, Jedidah M. Sakwa

**Affiliations:** ^1^ Radiology Department Kenyatta National Hospital Nairobi Kenya; ^2^ Department of Physics and Applied Physics University of Massachusetts Lowell Lowell MA USA; ^3^ Nuclear Engineering National Nuclear Regulator Johannesburg South Africa

**Keywords:** radiation dose, diagnostic reference levels, fluoroscopy, patient dosimetry

## Abstract

The purpose of this study was to assess the level of patient radiation dose received in general fluoroscopy examinations, compare the findings with the international diagnostic reference levels (IDRLs), and establish the initial institutional (local) LDRLs. A comprehensive survey was conducted for general fluoroscopy examinations using the medical records of a Radiology Department of a leading regional hospital over a period close to one year. The cumulative reference point air kerma $\left(K_{a,r}\right)$, kerma area product (KAP) and fluoroscopy time (FT) were recorded for six hundred and fifty (30% pediatric and 70% adult) patients undergoing routine fluoroscopy examinations using X‐ray equipment with built‐in integrated dose measuring system. Results which were obtained for adult general fluoroscopy indicated that 83% and 33% were below the IDRLs for KAP and fluoroscopy time, respectively. In children, 60% were found to be below the only available KAP diagnostic reference levels. Local diagnostic reference levels (LDRLs) have been proposed with respect to the missing DRLs for the K_a r_, KAP, and fluoroscopy time. The majority of the examinations in the study were performed with longer fluoroscopy time, patient dose values per examination type were found to be broad and the mean values above the international diagnostic reference levels. This calls for proper and improved training and radiation protection skills for the responsible personnel, especially the equipment operators.

PACS numbers: 87.53.Bn, 87.59.C‐, 87.59.cf, 87.53.Bn, 87.50.‐a, 87.53.‐j

## I. INTRODUCTION

The medical field over the years has benefited enormously from the use of X‐ray radiation with various new developments associated with diagnosis and therapy. The development of high X‐ray attenuating contrast media has resulted in diagnostic and therapeutic minimally invasive radiology.^(1)^ Most fluoroscopy procedures involve introduction of contrast medium through injection or infusion into the respective body part in order to identify existing congenital, acquired structural abnormalities or to assess organ functionality. The fluoroscopic procedures, therefore, cover all body regions with variation in fluoroscopy time and corresponding radiation doses, depending on the complexity of the procedure. In oncology patients, these procedures take a central role in diagnostic follow‐up assessment of treatment. Technological advances in fluoroscopy however, continue to increase, with significant benefits in medical patient care management. Although there is a low frequency of fluoroscopy investigations reported in the literature, the exposure time of these procedures can be long and involve high radiation exposure to the patient, as well as the equipment operators. The newer therapeutic clinical procedures are among the leading sources of medical radiation exposure.^2^, ^3^ A case of control study indicated a significant radiation effect on the prostate gland attributed to the diagnostic ionizing radiation during barium enema and hip examinations.^(4)^ Among the equipment dose management factors to consider are number of pulses per second, beam angulations, filtration, field of view size, and the number of images acquired, image quality in terms of contrast and resolution, the exposure time, and the focal point to skin distance.^5^, ^6^ A good understanding of equipment dose reduction factors by the operator allows for the optimization of patient radiation dose. These dose reduction factors present numerous radiation protection challenges in fluoroscopy, complicating the optimization of patient protection. The equipment radiation protection and dose reduction features must be well understood, properly applied, and checked regularly for optimal clinical image visualization. Some of the dose related factors are operator‐dependent and directly affect the level of patient radiation exposure. The KAP meters are appropriate for dose measurements, but difficult to use for assessing the levels of potential skin injuries.^(7)^ The “as low as reasonably achievable” (ALARA) principle has no defined limits, hence requires expert judgment from the equipment manufacturers and the operating personnel. Dose measurements like KAP and cumulative reference point air kerma (K_a,r_) lend support to the ALARA objectives. In developing countries typical of Kenya, the absence or minimal quality assurance and quality control, imaging guidelines, operator accreditation or licensing, X‐ray equipment in‐built dosimeters, local fluoroscopy protocols, and country specific DRLs^(8)^ bring about significant variation in fluoroscopy imaging techniques, indeterminate patient dose, and poor adoption of evidence‐based dose management practices. These quality assurance challenges are exacerbated by emerging health needs and lack of adequate resources coupled with the ballooning population. This study aimed to determine the radiation dose to patients, establish the necessary optimization strategies for fluoroscopy procedures, and compare the findings with the international DRLs.^2^, ^9^, ^11^, ^12^, ^13^, ^14^, ^15^

## II. MATERIALS AND METHODS

The study was undertaken at the Kenyatta National Hospital, a referral, teaching, and research hospital located in the capital city of Kenya, between June 2011 and April 2012. Frequency of fluoroscopic examinations was retrospectively analyzed from the patient records of the Radiology Department. The fluoroscopy examinations included water‐soluble upper gastrointestinal tract (GIT) contrast studies, barium meal, barium swallow (BaS), distal pressure colostogram, micturating cystourethrogram (MCU), hysterosalpingogram (HSG), and venogram. Barium Enema examination was not performed on this equipment during the study period. A C‐arm fluoroscopy unit (Philips, MultiDiagnost Eleva, Philips Medical Systems, Netherlands) connected to a high‐frequency generator (Optimus 50; Philips Medical Systems) was used in all the examination cases reported here. The X‐ray tube was positioned with the image intensifier at a variable source‐to‐image distance ranging from 95.5 cm to 125.5 cm. The control system for the angiographic equipment automatically sets the X‐ray exposure, kilovoltage (kVp), and tube current‐time product (mAs). The minimum tube filtration used was 2.5 mm aluminum in conjunction with additional copper filters of thickness 0.1 mm, 0.2 mm, 0.3 mm, and 0.9 mm. The fluoroscopy equipment utilizes digital high‐resolution continuous fluoroscopy with dynamic fluoroGrab (Philips Medical Systems) up to 30 images per second and acquisition speed of up to 8 images per second. The equipment has the following over‐couch II magnification modes sizes: 38 cm, 31 cm, 25 cm, 20 cm, and 17 cm. A built‐in, charge‐coupled device (CCD) camera is fitted in an image intensifier, and the examinations images obtained are acquired and saved in the machine operational computer in digital format. The fluoroscopy equipment unit used employed modern dose‐reduction techniques: adaptive measuring field, preset collimation, fluoroGrab, removable grid, iris/square shutters, intelligent exposure, automatic exposure control, digital subtraction angiography (DSA), adjustable acquisition speed, and last image hold (LIH).

Dosimetry information for both fluoroscopy and cinefluoroscopy was equipment‐generated for each examination using an integrated measuring system that displayed KAP (Gy.cm^2^), $K_{\text{ar}}$ estimates (mGy), and fluoroscopic time (minutes). These dosimetry systems are compliant with the dosimetry requirements set by the International Electrotechnical Commission (IEC).^(16)^ The K_a r_ indicated the total air kerma delivered to the interventional reference point (IRP). It did not include beam size (collimator position) or beam position with respect to the patient orientation (table position and gantry angulations). The location of IRP, according to IEC standards, is at the point along the central axis at 15 cm from the system isocenter toward the X‐ray focal spot.^(16)^ Calibration verification of the fluoroscopy equipment built‐in KAP meter was done by a medical physicist using an Unfors Xi solid state R/F detector (Unfors Instruments AB, Billdal, Sweden). The fluoroscopy examinations were performed by radiologists. For each examination case, the following parameters were recorded from the equipment computerized radiation information report: patient age/sex, examination clinical indication, $K_{a,r}$ (mGy), cumulative KAP reading (Gy.cm^2^), fluoroscopy time (minutes), number of runs (cinefluoroscopy or DSA acquisitions), total number of images of all the runs performed for a particular complete examination, field‐of‐view size, kVp, mAs, and the name of radiologist that performed the procedure. The cumulative KAP values were used with effective dose conversion factors to obtain effective doses for the full examinations.^(21718)^ Except for effective dose, which is not a measurable dosimetric parameter, the mean values obtained with respect to $K_{a,r}$, KAP, and fluoroscopic time were considered the LDRLs.

## III. RESULTS

### A. Fluoroscopy examinations

**Figure 1:**
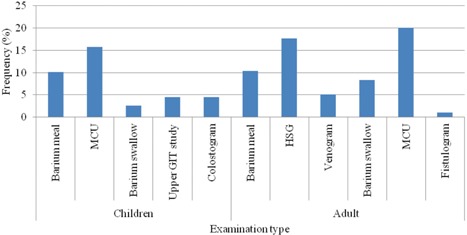
Frequency distribution of general fluoroscopy examinations considered in the study.

Figure 1 shows the annual fluoroscopy examination workload where 62% were adults and 38% children. In children, 26% of fluoroscopy examinations were MCU (16%) and barium meal (10%), and in adults the same two fluoroscopy examinations constituted 30%. HSG women examination constituted 18% of adult fluoroscopy examinations. Table 1 contains the indications for the fluoroscopy examinations in the study.

**Table 1:** Indications for general fluoroscopy examinations from the study

	*Examination*	*Indication*
Children	Barium meal	Pain and dyspepsia, investigate malrotation of gut, congenital anomalies.
	MCU	To demonstrate vesico‐ureteric reflux. Investigate stress incontinence and outflow tract obstruction. Urinary tract infection (UTI).
	Barium swallow	Assessment of tracheo‐esophageal fistulas, post‐surgical strictures. gastro‐esophageal reflux.
	Upper GIT Study	Congenital atresic segments look for signs of ulcers, cause of acid reflux disease, vomiting, or blood in the stools.
	Colostogram	Anorectal anomaly waiting colonic pull through surgery.
Adults	Barium meal	Dyspepsia, weight loss due to carcinoma, upper abdominal mass, gastrointestinal hemorrhage, partial obstruction, perforation.
	HSG	Investigation of blocked or twisted fallopian tubes, primary infertility, visualizes polyps or fistulas. To follow tubal surgery and recurrent abortions.
	Venogram	Acute deep vein thrombosis, unexplained edema, vein strictures/blockage and competence of valves and the site of incompetent perforating veins.
	Barium swallow	Dysphagia, assessment of left atrial enlargement, site of perforation, and preoperative assessment of carcinoma of the esophagus.
	MCU	To demonstrate vesico‐ureteric reflux and urethral strictures. Investigate stress incontinence and outflow tract obstruction (urethral strictures).
	Fistulogram	To investigate fistulas or sinuses between organs.

### B. Assessment of patient dose

Table 2 contains the distribution of exposure parameters for the fluoroscopy procedures considered in the study. The kVp and mAs values used in most of the examinations were indicative of the varying anatomic weight. Barium studies and MCU procedures were performed with the largest total number of images in both pediatric and adult patients. MCU procedures were performed with the largest mAs in adult patients, while the same was applied for barium meal and upper GIT studies in children. The duration of fluoroscopy time did not differ significantly among children for different examinations and age group, but the radiation exposure increased proportionally to the increase in their age/size. In fluoroscopic procedures, the three leading examination cases in fluoroscopy time for children were upper GIT, barium meal, and barium swallows; whereas in adult examinations, they were barium meal, venogram, and barium swallow. The nominal II magnification mode size that was selected in most examinations irrespective of patient age was found to be 38 cm.

Table 3 contains the patient radiation exposure values for complete fluoroscopic examinations. The leading three examinations with the largest radiation dose imparted to patients in decreasing order were barium meal, upper GIT studies, and barium swallow for children; similarly, barium meal, fistulogram, and MCU for adults.

**Table 2:** Mean (range) patient age, imaging parameters, and exposure factors for complete fluoroscopic examinations.

	*Examination*	*N*	*Age (yr)*	*Total Runs*	*Total images*	*FOV (cm)*	*kVp*	*mAs*	*Pulse Width (ms)*
Child	Barium Meal	19	1 (0−1)	11 (6−21)	13 (6−31)	38 (20−38)	70 (70−95)	4(1−23)	22(10−40)
		12	3 (2−5)	9(1−13)	11(1−30)	38 (20−38)	70 (70−77)	3 (1−6)	19 (6−31)
		10	9(6−10)	10 (7−12)	11 (8−19)	38 (20−38)	77 (73−77)	4 (1−7)	16(6−23)
		9	12(11−15)	9(5−12)	10 (5−19)	38 (20−38)	77 (77−102)	6 (3−10)	24 (13−38)
	MCU	15	1 (0−1)	10 (4−22)	14 (8−30)	38 (20−38)	66 (65−91)	3 (1−5)	22 (13−35)
		26	3(2−5)	10 (5−16)	16 (5−77)	38 (20−38)	70 (66−84)	2 (1−5)	20 (7−31)
		19	9 (7−10)	11 (7−19)	18 (7−56)	38 (20−38)	73 (65−75)	5(2−9)	22 (10−39)
		18	12 (11−14)	10(5−23)	17 (6−63)	38 (17−38)	74 (62−85)	8 (2−16)	35 (7−54)
	Barium Swallow	7	1 (0−1)	12 (3−21)	20 (3−46)	38 (25−38)	70 (65−81)	3 (2−5)	20 (6−32)
		6	3 (2−5)	8(5−9)	9 (5−12)	38(25−38)	70 (70−77)	3 (2−5)	17 (10−23)
	Upper GIT Study	12	1(0−1)	13 (8−21)	15 (8−31)	20 (20−38)	70 (70−71)	4(1−23)	21 (10−29)
		7	3 (2−4)	9(1−13)	12 (1−30)	38 (20−38)	73 (70−73)	4 (2−6)	24 (9−31)
		3	9 (7−10)	10 (8−11)	10 (8−11)	38 (20−38)	84(76−99)	6 (1−10)	20 (7−31)
	Colostogram	4	1(0−1)	7(5−10)	7(6−10)	38 (25−38)	77 (69−77)	2 (1−5)	14 (6−28)
		14	3 (2−4)	7(4−10)	7 (4−12)	38 (20−38)	78 (70−81)	2 (1−3)	10 (2−20)
		4	8 (6−9)	8 (1−13)	9 (1−14)	38 (20−38)	76 (70−81)	3 (2−6)	8 (7−10)
Adults	Barium meal	51	40 (16−80)	11(4−21)	13 (4−39)	38 (20−38)	102 (77−110)	8 (2−19)	34 (7−68)
	HSG	87	32 (21−49)	6(1−16)	8(3−21)	38 (20−38)	85 (75−105)	10 (3−23)	47 (6−75)
	Venogram	25	49 (21−74)	15(5−28)	16 (5−28)	38(17−38)	60 (55−85)	6(3−10)	24 (10−46)
	Barium swallow	41	48 (16−85)	10(3−25)	19(3−48)	38 (20−38)	80 (73−105)	5 (2−10)	20 (6−39)
	MCU	99	44 (17−92)	11(1−26)	16(1−70)	38 (17−38)	87 (73−109)	12 (4−27)	51(14−102)
	Fistulogram	5	40 (29−52)	9(6−13)	10 (7−13)	31−38(38)	90 (84−90)	12 (7−16)	49(10−77)

N=number of cases.

**Table 3:** Mean (range) patient dose parameters for complete fluoroscopic examinations, compared to dosimetric parameters in the literature.

			*Cumulative Reference Point Air Kerma (mGy)*		*Kerma Area Product (Gy.cm^2^)*			*Fluoroscopic Time (min)*			*Effective Dose (mSv)*		
	*Exam.*	*Age (yrs)*	$K_{a,r}$	$K_{a,r}$ *3^rd^Q*	*KAP*	*KAP 3^rd^ Q*	*DRL*	*FT*	*FT 3^rd^ Q*	*DRL*	*E*	*E 3^rd^ Q*	*E Values In Literature*
Child	Barium meal	0−1	9(3−26)	11	0.8(3.2−400)	1.2	2^b^, ^c^, 1,2^b^	4.9(2.2−6.5)	5.9	–	2 (3.2−400)	3	–
		2−5	17(2−41)	19	2.4 (3.2−400)	3	2^b^, ^c^, 1,2^b^	3.7(1.2−7.2)	4.4	–	1 (3.2−400)	2	–
		6−10	20(6−33)	27	3 (3.2−400)	4	4.5^b^, ^c^,2.4^b^	3.4(1.3−6.5)	4.0	–	3(1−5)	4	–
		11−15	27(13−43)	30	5 (2−9)	7	7.2^b^, ^c^,6.4^b^	3.8(1.4−8.0)	4.6	–	5(2−9)	6	–
	MCU	0−1	5(1−25)	10	0.5 (3.2−400)	2	1^b^, ^c^ 0.8^b^ 0.9^b^,	2.4(0.5−5.5)	3.5	–	0.8 (3.2−400)	2	–
		2−5	4(1−16)	10	0.6(0.1−2.1)	0.9	1.1^b^, ^c^, 0.8, 1.2^b^	2.5(0.4−19.1)	4.1	–	0.5 (3.2−400)	3	–
		6−10	14 (4−44)	20	2(0.6−5.2)	3	2.1^b^, ^c^ 1.5, 2.4^b^	3.3(0.6−11.6)	3.9	–	0.6 (3.2−400)	2	–
		11−15	23 (2−116)	30	4 (3.2−400)	6	4.7^b^, ^c^, 2.5^b^	2.5(0.5−8.6)	4.3	–	0.7 (3.2−400)	2	–
	Barium swallow	0−1	8(4−15)	11	1 (3.2−400)	1.8	1.5^b^, ^c^ ‐.1.3^b^,	3.9(1.1−7.1)	4.7	–	2 (3.2−400)	3	–
		2−5	8(4−12)	10	1(0.4−1.6)	1	1.5^b^, ^c^	3.2(2.0−4.5)	4.2	–	1 (3.2−400)	2	–
	Upper GIT Study	0−1	8 (3−15)	12	0.7(0.2−1.8)	1.1	–	4.7(1.5−8.1)	6.7	–	1 (3.2−400)	2	–
		2−5	23 (7−41)	40	3 (3.2−400)	6	–	4.8(3.4−6.5)	5.2	–	3 (3.2−400)	6	–
		5−10	20 (9−28)	30	2(1−7)	4	–	2.6(1.2−4.2)	3.3	–	2(1−4)	3	–
	Colosto.	0−1	4(3−5)	4	0.5(0.4−0.8)	0.7	–	1.7(0.5−3.5)	2.5	–	0.6(0.4−1.2)	1	–
		2−5	5(1−8)	6	1(0.2−2.9)	1	–	2.1(0.4−3.6)	2.9	–	0.8 (3.2−400)	2	–
		5−10	14 (6−23)	18	4 (2−7)	5	–	3.6(2.5−5.5)	4.5	–	1 (3.2−400)	2	–
Adult	Barium meal	16−80	61(3−272)	80	12(1−35)	15	13^b^, ^c^, 60^b^, ^d^, 17, 25^e^	3.3(0.4−11.1)	4.3	2.3^b^, ^c^2.7^b^	2 (3.2−400)	3	6 (3.2−400) ^f^, 2, 6.38,1.98^g^, 2.6^a^
	HSG	21−49	11(2−52)	15	2 (3.2−400)	3	2.9^b^, 4^c^	2.1(0.2−5.5)	2.6	1^b^, ^c^, 0.95^b^	0.6 (3.2−400)	2	1.2^a^
	Venogr.	21−74	16(4−43)	20	4 (1−8)	6	5^b^, ^c^ 7.5^b^, 9^b^	3.0(1.2−5.5)	3.5	2.3^b^, ^c^,2.2^b^,	0.4(0.1−2.2)	1	0.37^a^
	BaS	16−85	33 (3−123)	40	6(1−29)	9	9^b^, 11^b^, ^c^	2.8(0.2−7.5)	4.4	2.3^b^, ^c^	1 (3.2−400)	2	0.85(0.76−1.3) ^h^, 1.5^a^, ^g^
	MCU	17−80	39(6−175)	50	10 (3.2−400)	12	12^b^, 17^c^	2.4(0.5−8.3)	3.5	2.7^b^, ^c^ 1.9^b^	1.8 (3.2−400)	4	1.2, 2.4,3.7^a^
	Fistulo.	29−52	80(62−107)	90	23(14−31)	27	–	1.6(1.1−4.1)	2.5	3.8^b^	4(2−6)	5	2.65^g^,1.7^a^

^a^ Ref.#2

^b^ Ref.#9

^c^ Ref.#10

^d^ Ref.#11

^e^ Ref.#12

^f^ Ref.#13

^g^ Ref.#14

^h^ Ref.#15

Dash(–)=reference level not found.

## IV. DISCUSSION

### A. Fluoroscopy examinations

The use of fluoroscopy in patients for disease diagnosis and management is increasing due to technological advancement, availability of radiological equipment, and health care cost‐cutting measures. A study done some 22 years ago in Kenya found that 37,000 or 5.7% of total X‐ray examinations were general fluoroscopy;^(19)^ the present study, however, found that it constitutes 80,000 or 2.4% of all annual cases. The difference in the examination relative frequency between the two studies may be attributed to the introduction of newer imaging modalities such as the CT, ultrasound, and magnetic resonance imaging (MRI). The most frequently performed examination in both children and adults was found to be MCU and barium meal. The high frequency of HSG examinations were attributed to the young Kenyan population, with an increasing number of women attending the hospital fertility clinic.

### B. Imaging techniques and patient doses

The mean and range of the kVp in adult general fluoroscopy examinations were found to be larger but with lower mAs than the values reported in studies done in India and the Sudan.^20^, ^21^ In this study, the mean number of runs and images per examination category were comparable between children and adults. However, a comparison with the cited sources shows that the procedures in this study were performed with the acquisition of a larger number of runs and images. The general uniformity of long fluoroscopic time for most adult fluoroscopy examinations in this study compared to IDRLs indicates the need for proper training in, as well as the use of, optimized imaging techniques and protocols. The uniformity trend in radiographic imaging techniques for most examinations in this study is also suggestive of the potential for standardization of anatomical‐related imaging techniques and protocols. The standardization of the imaging techniques developed should address the number of runs and images acquired per radiologist for a complete procedure.

Also radiation safety in children is critical due to the greater chances of expressing radiation‐induced health effects over their lifetime.^(22)^ Additionally, optimization of imaging protocols in children will offset the potential increase in the radiation risks for the chronically ill children. The irradiated body region during the fluoroscopy investigation contains many of the most radiosensitive organs and tissues. In MCU examinations, the tissue and organs receiving considerable amounts of radiation dose include the urinary bladder wall, the lower large intestines, the small intestines, and the gonads. Considerable amounts of radiation during barium meal examinations are delivered to the stomach, the liver, the gall bladder, the adrenal glands, and the pancreas. The $K_{a,r}$ in children were smaller than those for adults due to the automatic selection of fluoroscopy technique triggered by the smaller patient size. The fluoroscopy examinations were performed with longer time and higher KAP, resulting in large effective doses (Table 3) that were also broad with respect to the measured values as compared to the available values in the literature.^2^, ^9^, ^10^, ^11^, ^12^, ^13^, ^14^, ^15^ The IDRLs for the $K_{a,r}$, KAP, and fluoroscopy time, especially in children, were not available in the cited literature for comparison.

The large patient radiation dose recorded was attributed to the prolonged fluoroscopic screening time, high number of runs, and the use of field sizes, as well as a higher frame rate than necessary. Patient protection may be improved by maximizing the distance between the X‐ray tube and the patient, minimizing the distance between the patient and the image receptor, avoiding exposing the same area of the skin in different projections, and minimizing projections through the thick body parts. Radiation dose to the patient is mainly dependent on the patient size, type of procedure, equipment used, and user experience. Radiologists should provide leadership to the imaging professionals in establishing local and national effective optimization mechanisms through the use of optimal fluoroscopic techniques, LDRLs, QA/QC records, and best practice quality improvement efforts. The practice should culminate into a regular review of applicable safety standards, LDRLs, development of professional accreditation guidelines, and continuous training and credentialing of specialists especially in pediatric imaging.

## V. CONCLUSIONS

Improved patient safety and adoption of standardized and optimized institutional protocols are possible to attain using equipment with an integrated dose system, as used in this study. The cumulative reference point air‐kerma data, along with KAP, should be routinely recorded in the patient records to enhance optimization in fluoroscopy practice and follow‐up for deterministic effects, if needed.

Patient dose is mainly dependent on the patient size, procedure, equipment used, and user experience. Radiologists should, therefore, play a pivotal role in establishing an effective optimization process through the establishment of institutional diagnostic reference levels (LDRLs) that will culminate in national diagnostic reference levels (NDRLs), development of professional accreditation guidelines, continuous training and credentialing of specialists. The scarcity of local specialists to offer fluoroscopy operational training programs may be addressed by collaborating with equipment manufacturers in order for them to develop computer‐based instructional materials that can be updated on regular basis or by exchange programs with well‐established institutions in the developed world.

## ACKNOWLEDGMENTS

We sincerely thank the Management and Radiology staff of Kenyatta National Hospital for accepting to participate in the International Atomic Energy Agency (IAEA) Project (RAF/9/033 — Strengthening Radiological Protection of Patient and Medical Exposure Control), and the IAEA for their support.

## Supporting information

Supplementary Material

Supplementary Material

Supplementary Material
