# Supplementary material for: Patient radiation exposure during general fluoroscopy examinations
Source: J Appl Clin Med Phys. 2014 Mar 6;15(2):262–70. doi: 10.1120/jacmp.v15i2.4555 (PMC5875479; doi:10.1120/jacmp.v15i2.4555)
Supplement: Supplementary file 1 — Supplementary Material [file ACM2-15-262-s001.rtf]

Figure 1: General Fluoroscopy examinations considered in the study. 
